# The Impact of Smoking on Long-Term Protection Following Hepatitis B Vaccination: A 24-Year Cohort Study

**DOI:** 10.3390/v16071137

**Published:** 2024-07-16

**Authors:** Marco Fonzo, Andrea Palmisano, Andrea Trevisan, Chiara Bertoncello

**Affiliations:** Department of Cardiac-Thoracic-Vascular Sciences and Public Health, University of Padova, 35131 Padova, Italy; andrea.palmisano@studenti.unipd.it (A.P.); andrea.trevisan@unipd.it (A.T.); chiara.bertoncello@unipd.it (C.B.)

**Keywords:** hepatitis B, vaccination, long-term protection, antibody concentration, HBsAb, anti-HBs, non-responder

## Abstract

The hepatitis B vaccination-induced immune response has been demonstrated to be associated with a number of factors, including age, sex, BMI, and the presence of comorbidities. Additionally, modifiable determinants such as smoking have been identified as influencing the response to vaccination. However, despite the evidence that smokers are at an increased risk of not responding to vaccination, the long-term effects of smoking on antibody persistence remain poorly understood. This study aims to assess the impact of smoking habits on long-term immunity following the primary vaccination cycle. Participants were required to have received a standard three-dose vaccine schedule in childhood, without subsequent doses, and to be between 18 and 24 years of age. Data on age, sex, BMI, age of administration of the first vaccine dose, and time between doses were collected. An antibody concentration < 10 IU/L was considered as non-protective. A total of 2133 individuals were included, 14.2% of whom were smokers. The mean age was 20.28 ± 0.92 years. The probability of having a non-protective antibody concentration was significantly higher in smokers than in non-smokers (AOR: 1.287; 95% CI: 1.002–1.652). The detrimental effects of smoking extend beyond the immediate effects on the vaccine response, also impairing the long-term immune response in individuals who received vaccinations during childhood.

## 1. Introduction

Since its introduction in the 1980s, the vaccination against the hepatitis B virus (HBV) has become a significant milestone in global prevention plans. The assessment of vaccine efficacy by follow-up and measurement of serum titre has made it possible to identify certain situations and factors in which vaccine response tends to be less than optimal. A number of factors potentially influencing individuals’ response to HBV vaccination have been studied. Some of these factors are related to the vaccination procedure itself, such as the anatomical site of injection, the dose administered, the type of vaccine (recombinant or plasma-derived), the route of administration, or the timing between doses. Others are associated with determinants at the individual level including sex, age, nutritional status, lifestyle, immunological status, ongoing comorbidities, or maternal HBV serological status at birth [1,2,3,4,5,6].

Among high-risk behaviours, tobacco smoking is a modifiable health determinant that has been found to be associated with a reduced response to the HBV vaccine in a number of studies [7,8,9,10]. However, as reported in a 2019 review [11], in addition to numerous studies confirming this association, there are a few in which this correlation was not found to be statistically significant [12,13,14,15].

As highlighted by the authors of the review, the evidence for some factors, such as smoking, psychological stress, and alcohol consumption, has not been unequivocal in the literature. This may be due to the fact that the vaccine response is a rather complex mechanism, in which many variables interact in a manner that is difficult to predict, thus impacting on the overall outcome and making it difficult to assess the actual weight of individual factors [11]. Furthermore, the possibility of confounding factors and inherent limitations of individual studies may also lead to inconsistent results regarding potential risk factors [15]. In another paper, it was suggested that the number of cigarettes smoked daily could be a further factor behind the discrepancy in the results between the various studies [16]. Indeed, in a previous study subjects who did not respond to vaccination were almost exclusively heavy smokers, defined as such on the basis of smoking 10 or more cigarettes per day [6].

Nevertheless, the role of smoking as a factor reducing the vaccine response is supported by a considerable number of studies. It is hypothesised that the cause lies, similar to obesity, in a state of chronic inflammation [16]. As early as 1987, Holt illustrated in his work some of the effects of tobacco on antibody production, such as a decrease in the IgG-mediated response and a suppression of the functioning of regulatory T lymphocytes [17]. A study investigating the impact of chronic immune activation reaffirmed how smoking can alter the immune system and, consequently, act pathogenetically by inducing its abnormal activity [18]. This corroborates previous findings that demonstrated an independent effect of tobacco use in influencing the lymphocyte response, resulting in reduced efficacy [8,19]. Conversely, nicotine in cigarettes also appears to play a role. By interfering with calcium-mediated intracellular cascades and antigen-mediated lymphocyte signalling pathways, this substance would negatively impact antibody production [10].

In light of these findings, it is evident that the impact of smoking on hepatitis B vaccine efficacy warrants further investigation. Smokers are at an increased risk of not responding to vaccination or responding weakly, and may therefore benefit from tailored vaccination strategies. These may include the administration of more doses in the baseline vaccination cycle, higher-than-standard doses, or more frequent recalls [10]. Additionally, the use of newer generation vaccines with higher immunogenicity, such as triple-antigen formulations, may be beneficial [16].

The impact of smoking on vaccine response has been primarily elucidated through investigations that assessed short-term immunity, which can be evaluated by monitoring antibody levels in the months following the conclusion of the primary immunisation cycle. As early as 1989, Shaw and colleagues observed that smoking was a variable independently associated with a diminished antibody response at the conclusion of immunisation cycles, in addition to age and BMI [8]. Winter and colleagues also demonstrated comparable outcomes by evaluating the seroconversion rate in a sample of healthcare workers vaccinated against HBV using two distinct schedules: one rapid (0–1–2–12 months) and one classic (0–1–6 months). In both groups, smokers exhibited lower antibody levels or, in some instances, a lack of response [9]. Conclusions similar to those presented here have been reached in previous studies evaluating short-term seroconversion rates following HBV vaccination [20,21,22]. 

In any case, a number of other variables must be considered in conjunction with the smoking habit in order to gain a full understanding of the phenomenon. For example, a study by Middlemann and colleagues found no statistically significant effect of smoking on vaccine efficacy in a sample of adolescent individuals [12]. The authors of this study suggest that this discrepancy with studies conducted on adults may be linked to a greater number of cigarettes smoked daily by adults, but above all to a longer period of time in which the individual was an active smoker. The interaction of smoking with the immune system would therefore only be noticeably manifested following prolonged exposure, which would thus act as a trigger to trigger a chronic pro-inflammatory state capable of interfering with the proper functioning of adaptive immunity [17,18].

A considerable number of studies in the literature indicate that tobacco smoking has a negative impact on the short-term response to HBV vaccination. However, the effects of smoking on the long-term persistence of vaccine-induced immunity have not yet been sufficiently assessed [23]. The aim of the present research is to investigate the impact of smoking habits on long-term vaccine protection, in particular for approximately 20 years following the end of the primary vaccination cycle carried out in childhood. The results of this study will facilitate a more comprehensive understanding of the role played by smoking as a modifiable risk factor, enabling the attribution of a specific weight to it on long-term antibody persistence. This, in turn, will facilitate the identification only on an anamnestic basis of subjects in whom the protection provided by the vaccine might be more at risk of being below the threshold. For such subjects, the use of a personalised vaccination schedule might be more appropriate.

## 2. Materials and Methods

We conducted a retrospective cohort study including healthcare students at the University of Padua, recruited at the time of the compulsory occupational medical examination for the start of hospital work or traineeships. The examinations were conducted between January 2004 and December 2020. Based on the Italian guidelines of the National Vaccine Prevention Plan (*Piano Nazionale di Prevenzione Vaccinale*, *PNPV*) in force at the time of examinations, anti-HBs antibody monitoring is routinely carried out for students and workers in the healthcare sector; participants were also tested for HBsAg and anti-HBc antibodies [24]. Inclusion criteria for the subjects were as follows: being born in Italy from HBsAg-negative mothers; having received a standard 3-dose vaccination course between 2 and 12 months of age, as witnessed by a certificate issued by the Public Health Office; not having received any booster doses afterwards; and being aged between 18 and 24 years at the time of inclusion in this study. The data collected were age, sex, body mass index (BMI), age at which the first dose of vaccine was administered, and the time interval between doses. The BMI variable was grouped into four categories: underweight up to a BMI of 18.49, normal weight between 18.5 and 24.9, overweight between 25.0 and 29.9, and obese from 30 and above. Considering the young age of the individuals enrolled in this study, active smokers and ex-smokers were merged into a single category for the purposes of evaluation.

A commercial chemiluminescent microparticle immunoassay (CMIA) was used up the end of the year 2017 to measure the concentration of anti-hepatitis B surface (s) antigens (anti-HBs). Afterwards, a different commercial kit was used, more specifically a chemiluminescent immunoassay (CLIA), LIAISON^®^ anti-HBs, manufactured by Sorin Group S.p.A. An antibody concentration higher than 10 IU/L was considered as protective, according to international standards [25]. However, it has to be said that many individuals with an anti-HBs concentration lower than 10 IU/L respond optimally to the booster dose, supporting the thesis that a strong immunological memory against HBV persists [26,27,28]. In the light of this, some argue that the use of a different threshold should be considered [28,29,30]. 

Descriptive analysis was performed using the mean and standard deviation for continuous variables and absolute and relative frequencies for discrete variables. To assess differences in the distribution of discrete variables, Pearson’s Chi-square (χ^2^) test was used, while for continuous variables the Student’s t-test with Bonferroni correction was employed; *p*-values and 95% confidence intervals (CIs) were calculated. To assess the effect of smoking on the chance of having an antibody concentration below the threshold, the odds ratio, both crude and adjusted for participant age, age at first vaccine dose, time between doses, and nutritional status, was calculated using one-step logistic regression analysis; *p*-values and 95% CIs are reported. IBM SPSS version 28 was used for statistical analysis. This study is the result of findings from the data collected during the occupational medical examination of healthcare personnel. Consequently, it was not necessary to submit this study for evaluation by an ethics committee. The subjects undergoing the health surveillance visit, in any case, signed a document authorising the processing and possible publication of aggregated data in a strictly anonymous form. The data were collected in accordance with the principles of the Declaration of Helsinki, in accordance with the national legislation in force at the time of collection and in compliance with data protection regulations.

## 3. Results

This study included a total of 2133 participants (Table 1).

They were all vaccinated with a three-dose schedule between May 1990 and August 2003. None of the participants tested positive for HBcAb or HBsAg. Overall, the mean age was 20.28 ± 0.92 years and 62.6% of the study population was female. A proportion of 14.2% of participants (302/2133) were smokers, either current or former smokers.

In smokers, the proportion of females was significantly lower than in non-smokers (53.3% vs. 64.2%, *p* < 0.01). The mean age at the time of analysis and at the time of first vaccination was slightly higher in smokers (20.56 ± 1.11 vs. 20.24 ± 0.87, *p* < 0.01 and 3.05 ± 0.86 vs. 2.92 ± 0.58, *p* < 0.01, respectively), whereas no significant difference was observed in the timing of vaccination. In addition, smokers were slightly more overweight/obese than non-smokers (12.2 vs. 9.2%, *p* < 0.05).

The overall proportion of individuals with an antibody concentration lower than 10 IU/L was 51.3% (1094/2133), with a difference between non-smokers and smokers (50.6% vs. 56.6%, *p* = 0.103, respectively). The probability of having an antibody concentration of less than 10 IU/L was assessed using one-step binary logistic regression (Table 2).

While the crude OR was 1.225 (95% CI: 0.959–1.565), after adjustment for sex, age at analysis, age at first vaccine dose, timing of vaccine administration, and nutritional status, the AOR was 1.291 (95% CI: 1.006–1.657).

## 4. Discussion

The results of this study provide further support for the hypothesis that the habit of tobacco smoking may have a substantial effect on the long-term persistence of antibodies. The analysis revealed a statistically significant association between smoking and an increased chance of having an anti-HBs titre below the safety threshold, with an adjusted odds ratio (AOR) of 1.287 and a 95% confidence interval (CI) of 1.002–1.652. This association was observed in comparison to the non-smoking population. One can approximate the value to an increased probability of having a sub-threshold antibody concentration of approximately +29%. The results emerging from this study rank alongside the existing literature on the negative influence of smoking on the short-term response to the hepatitis B vaccine [8,9,20,21], and also prompt further reflection on the potential for a heightened risk of temporally more limited vaccination coverage in a specific population group.

The impact of smoking as a negative immunomodulatory agent was well described by Holt in his aforementioned 1987 study, which highlighted how the effect of smoking on the immune system causes a reduction in the efficacy of the IgG-mediated antibody response and a suppression of normal T lymphocyte function [17]; Younas and colleagues have also reiterated how aberrant immune activation is directly attributable to habitual exposure to cigarette smoke [18]. A certain role is also played by the nicotine contained in cigarettes, which appears to act as an interfering agent in the intracellular signalling cascades that lead lymphocytes to mediate antibody production [10].

In addition, it should be noted that the effect of smoking can also affect vaccinations other than HBV vaccination in a variety of ways: as pointed out by Zimmermann and Curtis in a review of the literature on factors influencing vaccine outcomes [11], the impact of smoking on human papilloma virus (HPV) vaccination does not appear to have a statistically significant effect on antibody titres induced by the vaccine response, but is associated with an increased risk of presenting low avidity antibodies to the vaccinated antigen [11,31]. In addition, some findings suggest that smoking is correlated with higher rates of seroconversion after administration of the live attenuated influenza vaccine (LAIV) vaccine, but with faster antibody titre decay when compared to non-smokers, in both the LAIV and the trivalent inactivated vaccine (TIV) [11,32]. Smoke-induced alterations on HBV vaccination are therefore likely to act on several fronts: affecting the cell-mediated immune response and its role in developing an effective post-vaccine immunogenic response, causing a more rapid decay of the antibody titre in the long term, and potentially also inducing a decrease in avidity in the generated antibodies.

Overall, the effect of smoking as an interfering agent on the immune response can be compared to a certain extent to that of obesity. Obesity is an important and validated risk factor for a reduced response to HBV vaccination [8,10,33] due to a pro-inflammatory action mediated by adipose tissue itself, which will consequently be more pronounced as the degree of obesity increases [16,34]. It can be assumed that, similarly to what happens in a context of obesity, in the long term the action of smoking may also lead to a set of systemic pro-inflammatory changes and abnormal immune activation. Similarly, the chronic smoke-mediated effect on the immune system could conceivably cause a depletion of the lymphocyte cellular component involved in immune response and antibody production, with an increase in B lymphocytes with an ‘exhausted’ phenotype in a manner quite similar to what occurs in patients with a high BMI [35].

Seroprotection against HBV is defined as the presence of an anti-HBs antibody concentration ≥ 10 IU/L following a course of vaccine immunisation or as an outcome of the resolution of a wild-type HBV infection [36]. The ideal vaccine should act by producing a sufficient titre as quickly as possible, while remaining effective over the long term and providing a level of protection comparable to that of individuals who have successfully resolved the infection [37]. Data show that, following administration of a standard three-dose vaccine cycle, more than 95% of healthy recipients develop an adequate serological response with an anti-HBsAg concentration ≥ 10 IU/L [38].

Generally speaking, the antibody concentration induced by vaccination ranges from non-responders, who do not reach the cut-off of 10 IU/L, to subjects with an adequate response of even more than 10,000 IU/L; in order to reach an antibody level ≥ 10 IU/L one or two months after the end of the immunisation cycle, at least three doses injected at a temporal distance from each other are required; the antibody level, although decreasing rapidly within a year after the last dose, tends to decline more slowly after the year [37]. 

Although antibodies tend to decrease, the effectiveness of the vaccine seems to be maintained over a long period of time, potentially even after 20–30 years: a 2019 study showed that in adult subjects who had been vaccinated for two or three decades a concentration above the minimum cut-off could be detected in about 90% of the participants, even before a booster dose was administered. The latter was also able to induce a correct response from the memory cells, thus highlighting the long persistence of effective immunisation even without the need for booster doses [39].

In any case, a finding of an anti-HBs concentration below the threshold set by international guidelines (10 IU/L) is not necessarily indicative of a lack of protective response to infection: an active role is played by memory cells, which, if adequately present and functioning, enable an adequate response to be generated rapidly. As proof of this hypothesis, several studies show that even in subjects with an antibody level below 10 IU/L a good anamnestic response is generally triggered by the administration of a booster dose [40,41,42]. This suggests that the HBV vaccine may confer long-term protection irrespective of a decrease in circulating anti-HBs titre, which would then be an analytical finding not necessarily related to a true loss of protection; therefore, in healthy and immunocompetent individuals, the administration of booster doses in addition to the primary course is not currently indicated. The injection of a booster dose may however be considered for subjects with anti-HBs < 10 IU/L and belonging to risk categories. However, the long-term declining kinetics of the antibody level may also imply an actual loss of the protection conferred by the vaccine years earlier and expose one to infectious risk, as shown in two studies carried out on subjects vaccinated at birth in which a percentage of individuals expressing anti-HBc antibodies, serological markers of a past or present infection, were found [43,44].

In the light of the evidence available to date, it can be concluded that, although the coverage induced by the HBV vaccine is supposed to be long-lasting and effective even without high levels of circulating antibodies due to immunological memory, its protective effect may drop over the long term and expose one to a certain risk of infection. In individuals vaccinated at birth and belonging to at-risk groups, such as healthcare workers, a booster dose may be useful to restore adequate protective capacity [45,46].

The first strength of this investigation lies in the study design. This work was set up as a longitudinal cohort study, evaluating a rather extensive time span and thus ensuring, over approximately 20 years of data collection, the formation of an extremely large database from which to draw for the evaluation and analysis of long-term antibody persistence in relation to multiple potentially influential variables. The use of the long-term protection conferred by vaccination against HBV as an outcome is a second important strength of this study: with regard to the impact of cigarette smoking on vaccination, there is a large number of studies in the current literature analysing the influence of this risk factor on immunogenicity and short-term protection. On the other hand, the persistence of anti-HBs antibodies in the long term, especially in relation to the influence of smoking, is an area that has been little explored to date, with a relatively small number of studies on this subject. As far as the methodological part of this work is concerned, a major strength lies in the sample size used, due to the extensive size of the database created in the time interval used for data collection; finally, this study relied on a relatively small number of variables considered, but it was characterised by a high degree of completeness, due to the rigorous data collection framework used.

A first limitation of this study is related to the impossibility of tracing the precise type of vaccine received by the sample subjects. However, considering the age range analysed and the time period in which the data pool was collected (from January 2004 to December 2020), it can reasonably be assumed that the subjects were vaccinated with recombinant formulations: the transition from plasma-derived vaccines to recombinant vaccines in Italy occurred gradually around the mid-1980s. At that time, the main recombinant vaccines available and most widely used were Engerix-B (10 μg) and Recombivax HB (5 μg). The latter was used particularly in the paediatric age group, as was the case of our study population [24,47].

A second limitation of the present study is the lack of data concerning the amount of cigarettes smoked daily by the smokers analysed, as well as the absence of information concerning the length of time the person smoked; no analytical data are available concerning possible discrepancies between subjects who can be defined as heavy smokers and others who can be classified as light or occasional smokers, just as no potential differences in association with subjects who were smokers for a longer or shorter time emerged. In addition, current and former smokers were combined into a single category. However, participants were all young subjects (average age of around 20 years), thus making any adjustments on the basis of being or having been a current smoker virtually irrelevant for the purposes of this study. It may also be assumed that there has not been a sufficiently long time for the full manifestation of the negative effects caused by smoking, although an increase of approximately 29% in the chance of a sub-threshold anti-HBs concentration is already a particularly relevant finding within the smoking sub-population, considering the relatively short life span in which these persons were exposed to the risk factor.

A further limitation is the lack of data on BMI during childhood and adolescence. Although it should be noted that obesity is a phenomenon related to an interaction between environmental, behavioural, and genetic factors, by virtue of a certain predisposition to obesity, it can be assumed that a high adult BMI often, though not always, reflects a similar condition in childhood, with an estimated five-fold higher risk in obese children and adolescents of also being obese as adults [48,49] (Simmonds et al., 2016).

Finally, another potential limitation is the lack in our sample of individuals with any comorbidities that could affect vaccine efficacy or long-term antibody persistence. It is noteworthy that a study conducted in Italy revealed that in individuals with coeliac disease who had been vaccinated against hepatitis B as adolescents the seroprotection rates after approximately a decade were significantly lower than in a healthy reference sample. The geometric mean concentration (GMC) of the coeliac group was 29.38 mIU/mL, while that of the healthy individuals was 250.6 mIU/mL (*p* < 0.001) [50].

## 5. Conclusions

Previous research has indicated that smoking has a detrimental effect on the immediate immune response to HBV vaccination in adult populations. This study has demonstrated that tobacco smokers are more likely to have a protective antibody concentration below the recommended threshold than non-smokers up to two decades after the primary course of vaccination in childhood. Therefore, smokers can be considered to be at an increased risk. Given the high protective efficacy of the vaccine, the prevalence of smoking from a very early age, and the possibility of successfully and rapidly raising antibody titres by revaccination, individuals with a history of smoking could potentially benefit from initiating personalised antibody screening and possible vaccine booster doses to re-establish an adequate protective threshold in the event of a temporally accelerated decline in circulating anti-HBs levels.

## Figures and Tables

**Table 1:** Characteristics of the study population by smoking habit.

		Non-Smokers (*n* = 1831)		Smokers (*n* = 302)		Total (*n* = 2133)		
		*n*	%	*n*	%	*n*	%	*p*-Value
Female		1175	64.2%	161	53.3%	1336	62.6%	**
Age (years; mean, SD)		20.24	0.87	20.56	1.11	20.28	0.92	**
Age at 1st dose (months; mean, SD)		2.92	0.58	3.05	0.86	2.94	0.63	**
Time 1st–2nd dose (days; mean, SD)		57.38	18.72	58.00	25.00	57.47	19.73	
Time 2nd–3rd dose (months; mean, SD)		7.20	1.15	7.14	1.37	7.19	1.18	
BMI	Normal	1453	79.4%	245	81.1%	1698	79.6%	*
	Overweight	153	8.4%	33	10.9%	186	8.7%	
	Obese	14	0.8%	4	1.3%	18	0.8%	
	Underweight	211	11.5%	20	6.6%	231	10.8%	

* <0.05; ** <0.01.

**Table 2:** Probability of an anti-HBs concentration below 10 IU/L. Crude and adjusted odds ratios, 95% confidence intervals, *p*-values.

	Crude OR	95% CI	*p*-Value	Adjusted OR *	95% CI	*p*-Value
Smokers (vs. non-smokers)	1.225	0.959–1.565	0.104	1.291	1.006–1.657	0.045

* The odds ratio was adjusted for all variables shown in Table 1.

## Data Availability

Data are contained within the article.
